# A Comprehensive Review on Chemical and Pharmacological Potential of *Viola betonicifolia*: A Plant with Multiple Benefits

**DOI:** 10.3390/molecules24173138

**Published:** 2019-08-29

**Authors:** Komal Rizwan, Shakeel Ahmad Khan, Ikram Ahmad, Nasir Rasool, Muhammad Ibrahim, Muhammad Zubair, Hawa ZE Jaafar, Rosana Manea

**Affiliations:** 1 Department of Chemistry, Government College University, Faisalabad 38000, Pakistan; 2 Department of Chemistry, Government College Women University, Faisalabad 38000, Pakistan; 3 Center of Super-Diamond and Advanced Films (COSDAF) and Department of Chemistry, City University of Hong Kong, Hong Kong, China; 4 Department of Applied Chemistry, Government College University, Faisalabad 38000, Pakistan; 5 Department of Environmental Sciences and Engineering, Government College University, Faisalabad 38000, Pakistan; 6 Department of Crop Science, Faculty of Agriculture, University Putra Malaysia, Serdang 43400, Selangor, Malaysia; 7 Faculty of Medicine, Transilvania University of Brasov, 500036 Brasov, Romania

**Keywords:** *Viola betonicifolia*, antioxidant, isolated, bioactive constituents, nutrients, antipyretic, extraction, techniques

## Abstract

*Viola betonicifolia* (Violaceae) is commonly recognized as “Banafsha” and widely distributed throughout the globe. This plant is of great interest because of its traditional, pharmacological uses. This review mainly emphases on morphology, nutritional composition, and several therapeutic uses, along with pharmacological properties of different parts of this multipurpose plant. Different vegetative parts of this plant (roots, leaves, petioles, and flowers) contained a good profile of essential micro- and macronutrients and are rich source of fat, protein, carbohydrates, and vitamin C. The plant is well known for its pharmacological properties, e.g., antioxidant, antihelminthic, antidepressant, anti-inflammatory, analgesic, and has been reported in the treatment of various neurological diseases. This plant is of high economic value. The plant has potential role in cosmetic industry. This review suggests that *V. betonicifolia* is a promising source of pharmaceutical agents. This plant is also of significance as ornamental plant, however further studies needed to explore its phytoconstituents and their pharmacological potential. Furthermore, clinical studies are needed to use this plant for benefits of human beings.

## 1. Introduction

Demand of medicinal plants is increasing day by day around the globe for improved quality of life [1,2]. In recent times, investigation on plants to find out and isolate plant-based natural compounds is getting much attention by scientist worldwide. Numerous corroborations have been perceived in different reports that indicate significant potential towards their medicinal use. Plants are widely exploited in numerous traditional systems (Ayurveda, Siddha, and Unani) to combat different kinds of ailments [3]. Medicinal plants are considered the chief fount for biodynamic compounds that have analeptic value [4,5]. Plants are also rich source of fodder, fuel, and timber. Several kinds of commercially available synthetic drugs to cure different types of maladies have many side effects. Therefore, the demand for herbal medicines is increasing because they are safe, economical, effective, and free from side effects. Moreover, herbal remedies also provide a cure for certain age-related diseases, such as memory loss, osteoporosis, etc. [6]. 

The family Violaceae is also known by different names such as Retrosepalaceae, Leoniaceae, and Alsodeiaceae. This family includes ~20 genera as well as 800 species [7]. Viola is largest genus of the family Violaceae. This genus is comprised of 500 species, which are distributed worldwide. In Pakistan, the family Violaceae is signified through only one genus named as Viola which have 17 different species [8], among which some are perennial and annual herbs consisting of short-stem and rhizome, but the latter one is present or absent in some species. This family is of little economic importance [8]. Traditionally, many species of this genus have been patronizing in various ailments from ancient times, among which some of them have been earlier authenticated scientifically, such as antifungal, antibacterial, antioxidant, antihypertensive, antipyretic, antidyslipidemic, anti-HIV, analgesic, anti-inflammatory, antiplasmodial, diuretic, anthelmintic, anticancer, antiasthmatic, antifebrile, etc. [9,10]. In Chinese traditional medicines, plants of this genus are used as detoxicating agents and for the treatment of severe pyogenic infections, e.g., carbuncles, furuncles, and boils [10]. 

Some of the species of this genus are used to treat appendicitis, acute nephritis, hepatitis, snake bites, and bronchitis. Many species of this genus are exploited as traditional remedies in Russia, Korea, Romania, and Iran for the treatment of trophic ulcers, shingles, and diuretics and, while as anti-inflammatory agents for skin eruptions, rheumatism, bronchitis, and dermatitis in traditional medicines. The plants of this genus are used in landscape beautification because of splendid colors of flowers [10]. This genus is rich source of natural compounds having different structures. Almost 200 natural compounds have been identified and isolated from various species of viola including terpenoids, flavonoids, amides, sterols, saccharides, essential oils, aromatic compounds, and other derivatives [11].

As part of our studies of documenting the indigenous flora of Pakistan [12,13,14,15,16,17,18,19,20,21], to our best knowledge, no review has been reported yet covering the valued features of *V. betonicifolia* comprehensively. It is a nutritive plant, and due to widely exploitation in folk medicine, enthused us to write review on this medicinal plant. We have comprehensively described its distribution, nutritional, and medicinal properties, together with its phytochemicals.

## 2. Methods 

Extensive literature survey was carried out with different keyword as *Viola betonicifolia*, nutrients, viola, antioxidant, antimicrobial, volatile, etc., to search electronic databases such as PubMed, Web of Science, Scopus, Science Direct, Scifinder, Google, and Google Scholar for information. Different relevant abstracts and all full text articles and books were consulted.

## 3. Morphology, Nutritional, and Traditional Values of *Viola betonicifolia*

**Figure 1:**
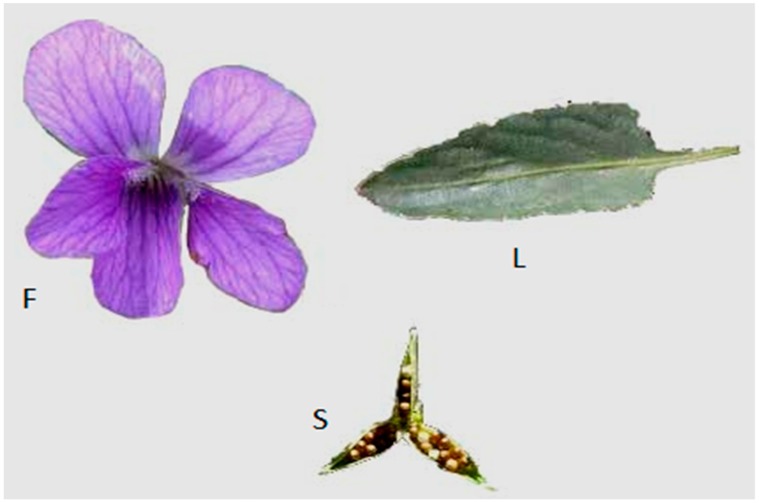
Different parts of plant *V. betonicifolia*: L., leaf; F., flower; S, seed.

The plant *Viola betonicifolia* (Genus: Viola, Family: Violaceace) is recognized as Banafsha or Banfosha in Pakistan. It may be found in different habitats, including woodland, forest, shrubland, and herb fields [22]. It is a perennial herb and its height is 8 to 20 cm. Distinguishing characteristics of this species are that it is longer in length, slim, has arrow-shaped-leaves, which usually enlarge from the base, possesses no stem, and is 6 cm (2.4 in) in length with a v-shaped sinus at the base. These are habitually brighter and have fresh-green color (Figure 1). The length of Lamina is mostly 1–8 cm with a width of 5–25 mm. The leaf margins of this plant are entirely or marginally serrate. 

The linear stipules are present which are fused to petiole, and may be entire or laciniate. The sepals are 3.5–7 mm long. The color of the petals is violet, but can also be whitish. Petals are 8.5–14 mm long. The plant taste is little bit piquant and have spice fragrance [23]. Seeds are rounded-ovate, smooth, and shiny [24]. 

Its attractive bright purple flowers, which are 1–1.5 cm in diameter, usually bloom in summer and spring. The flowers of this plant are shadowed through small pale brown pods with tiny blackish seeds. The roots and rhizome are slender, unbranched, and short, respectively. The flowering season of this plant is from September to January. It usually provides ground cover for butterflies and is a known food plant of the butterflies. *V. betonicifolia* exists in several countries over the globe, including Pakistan, Nepal, India, China, Sri Lanka, Burma, Malaysia, and Australia (Flora of Pakistan) [9]. In Pakistan, this plant possess habitat in northeastern as well as northwestern regions (Swat, Hazara, and Dir). Conventionally, this plant was exploited as diaphoretic, purgative, astringent, anticancer, antipyretic, and to treat various diseases such as epilepsy, nervous disorders [25,26], cough [27], skin disorders, sinusitis, blood disorders, pharyngitis [28], kidney diseases, pneumonia, and bronchitis. The leaves and flowers of this plants are widely used for the prevention of many health issues including skin infections, blood disorders, cough, sinusitis, pharyngitis, and lung troubles [28,29], while the fruits and roots are employed for treatment of pneumonia, kidney diseases, and bronchitis problems. On the other hand, the leaves are also valuable for treating boils [29]. 

### 3.1. Microscopy and Physicochemical Characteristics of V. betonicifolia

The physical parameters of *V. betonicifolia* are mostly constant and pharmacognostic investigations of these factors are supportive in setting standards for a crude drug. The various already determined characteristics of this plant (veinlet termination number, vein islet number, epidermal cell number, stomata number, and palisade ratio) are reported in Table 1 [23]. These parameters are very important in evaluating the crude drug and preclusion of some contamination. Consideration of moisture content is highly important for protecting the crude drug from microbial growth. The drug is highly stable in lower moisture contents, thus the chance of microbial growth will be less: more moisture will enhance the growth of microbes. Lower moisture content also increases the shelf-life of the drug [30]. The dried form of *V. betonicifolia* is usually anticipated to possess a longer shelf-life with lower chance of microbial growth because of its lower moisture content (5.2%). Mean ash values (%) was found to be 9.8 (total), 1.05 (acid-insoluble ash) and 0.7 (water-soluble-ash). The total ash value was found to be comparatively low, and this might be because of the low concentration of inorganic compounds. The ash values are important quantitative standards and are helpful in determining the purity and authenticity of drug. Acid insoluble-ash values are useful and they indicate higher digestibility value when the plant is being used up.

### 3.2. Extractive Values of V. betonicifolia with Different Solvents 

Muhammad et al. [23] extracted *V. betonicifolia* with six different solvents. The highest extract yield was obtained with methanol (18% *w*/*w*), followed by hexane (11% *w*/*w*) and water (8.5% *w*/*w*). Whereas the yield of chloroform extract was 4% *w*/*w*, ethyl acetate extract 3% *w*/*w*, and butanol extract 5% *w*/*w*. Plant extracts are good therapeutically in comparison to direct plant exploitation, this is why the choice of solvent has a large impact on achieving a worthy extract yield. Therefore, to achieve good extraction yield of *V. betonicifolia*, the best solvents would be *n*-hexane and methane. 

### 3.3. Nutritional Values of V. betonicifolia

Macronutrients like calcium (Ca), potassium (K), and sodium (Na) and micronutrients like cobalt (Co), cadmium (Cd), zinc (Zn), iron (Fe), nickel (Ni), copper (Cu), chromium (Cr), and lead (Pb) were explored in *Viola betonicifolia* (whole plant) and its different solvent fractions by employing flame photometry and atomic absorption spectrophotometer, respectively. It was anticipated that cadmium and cobalt were not identifiable in the investigated testers; however, on the other hand, all remaining nutrients existed in the tested samples, but in varying concentrations. The concentrations of micro- and macronutrients found in the extracts were compared with the threshold limit for the plants and their per diem consumption was estimated on base of dosages such as 15 g/70 kg/day or 214 mg/Kg bw, advised by hakims in their practice. Surprising results were anticipated for the metal chromium, whose concentration was more than the threshold limit in all the investigated tasters. Furthermore, plant powdered material was examined for its proximate analysis of which results were indicated that this plant is a potential source of life essential nutrients such as vitamin C (150 mg/100 g), fibers (39.01%), carbohydrates (21.42%), proteins (15.70%), and fats (18.70%). Henceforth, *Viola betonicifolia* is an admirable source of numerous macro- and micronutrients for human being consumption and could be employed as a nutritious supplement [22]. From the various macronutrients, Ca, Na, and K are important for various functions of the human body, as calcium performs a substantial part in skeletal mineralization, blood coagulation, and maintenance of normal tone. Furthermore, calcium also regulates the excitability of skeletal muscle, stimulated through the secretion of exocrine glands, and is involved in preserving cell membrane integrity as well as permeability [31], specifically in relation to sodium and potassium exchange. Sodium and potassium are both involved in the transmission of nerve impulses and control many other biological functions of the human body [32]. The considerable amount of these macronutrients was found in plant *V. betonicifolia*; similarly, micronutrients are needed by human beings at all stages of life as there are many synthetic supplements nowadays are commercially available. However, people are still pursuing macronutrients based on natural sources because of the causative adverse effects of synthetic drugs, the plant *V. betonicifolia* was found to have significant amount of important micronutrients and macronutrients (Table 2).

## 4. Phytochemistry

Various reports demonstrated that the extract of the whole plant *V. betonicifolia* in methanol solvent is a potential and substantial source of several phytochemicals including alkaloids, proteins, flavonoids, tannins, phenolic compounds, saponins, and sterols, as well as triterpenoids [33,34,35].

**Figure 2:**
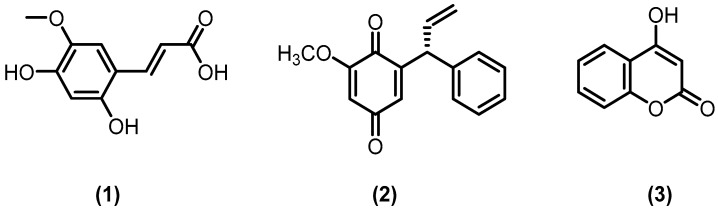
Isolated bioactive constituents of *V. betonicifolia*: 2,4-dihydroxy, 5-methoxy-cinnamic acid (**1**), 3-methoxydalbergione (**2**), and 4-hydroxy coumarin (**3**).

The new derivative of cinnamic acid in the form of off-white needles was sequestered from the whole plant of *V. betonicifolia* [36] and its structure was elucidated as 2,4-dihydroxy,5-methoxy-cinnamic acid by the exploitation of numerous spectroscopic techniques as High-resolution electron impact mass spectrometry (HREI-MS) and 1D and 2D NMR (Figure 2, **1**). The isolated derivative of cinnamic acid was evaluated for antioxidant activity of which results were demonstrated that DPPH (diphenyl-2-picryl hydrazyl) free radicals were significantly scavenged by it through the exhibition of IC_50_ = 124  ±  5.76 µM, which was further compared with standard employed antioxidants such as Vitamin C (IC_50_ = 90  ±  0.56 µM) and α-tocopherol (IC_50_ = 96  ±  0.46). Moreover, the isolated compound was found to exhibit modest antiglycation propensity (IC_50_ = 355  ±  7.56 µM) as comparable to that of the employed standard rutin (IC_50_ = 294  ±  0.56 µM) but, it was perceived that the isolated new derivative of cinnamic acid was nonhazardous to PC-3 cell line. Thus, it was corroborated that the new isolated derivative of cinnamic acid possesses significant antiglycation potential, which was enhanced because of its antioxidant potential and, consequently, demonstrated an ideal natural therapeutic option for the management and treatment of diabetes very effectively and impressively. 

Muhammad and coworkers [37] sequestered a new compound—3-methoxydalbergione (Figure 2, **2**)—from the collective ethyl acetate factions as well as chloroform fractions of whole plant of *V. betonicifolia*, in the form of yellow spongy mass. This compound significantly inhibited urease. This compound showed 82% inhibition at 0.5 mM (IC_50_ = 169 ± 2 μM). The urease enzyme assay and docking studies of this compound showed that this may be considered as the lead motif for urease inhibiting drugs.

**Figure 3:**
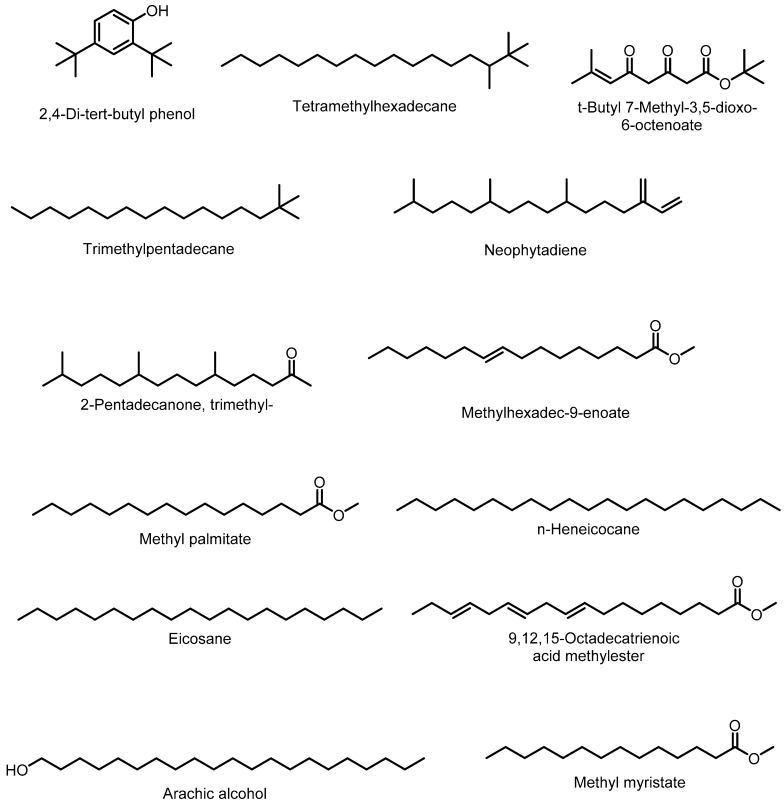
Major components identified in volatile oils (subfractions F1–F6) of *V. betonicifolia*.

4-hydroxy coumarin (Figure 2, **3**) in form of white powder was sequestered from combined chloroform and ethyl acetate fractions of *V. betonicifolia* whole plant [38]. This compound was found safe in acute toxicity test up to the dose of 150 mg/kg. Furthermore, while performing the traction and chimney tests, 4-hydroxy coumarin demonstrated substantial and enhanced muscle relaxant effects, which were more pronounced with increasing concentration (dose-dependent manner), while this compound showed no sedative and hypnotic potentials.

## 5. Pharmaceutical Potential of *Viola betonicifolia*

*Viola betonicifolia* is medicinally very important, and in this section the pharmacological importance of this plant is comprehensively described (Table 3).

### 5.1. Antimicrobial Activity

The antimicrobial propensity of the crude methanolic extract and different fractions (*n*-hexane, chloroform, ethylacetate, *n*-butanol, and aqueous) of whole-plant *V. betonicifolia* was checked against seven bacterial strains: *Escherichia coli*, *Bacillus subtilis*, *Shigella flexeneri*, *Staphylococcus aureus*, *Pseudomonas aeruginosa*, and *Salmonella typhi*. Plant extracts and fractions were found inactive against *S. aureus*. The chloroform fraction showed potent inhibition (20 mm and 17 mm) against *E. coli* and *S. typhi*, respectively. The *n*-hexane fraction did not show activity against all bacterial strains. All fractions showed considerable activity against tested bacterial strains. Antifungal activity of all fractions was checked against various fungal strains (*Trichophyton longifus*, *Candida albicans*, *Fusarium solani*, *Aspergillus flavus*, *Microsporum cani*, and *Candida glabrata*). All extracts showed low-to-moderate activity. The methanolic extract was found active only against *T. longifus* and *M. Cani* with 30% and 20% inhibition, respectively. The *n*-butanol fraction was found inactive against all fungal strains. Chloroform and ethyl acetate fractions inhibited the growth of *M.* cani (20 and 30%) and *F. solani* (7 and 10%), respectively. The aqueous fraction inhibited the growth of *C. albicans* (30%) and *M. cani* (40%). Miconazole and amphotericin B were used as standard drugs [39].

Muhammad et al. [40] reported the antimicrobial activity of various subfractions of plant *V. betonicifolia*. These subfractions (F1–F5) were obtained from column chromatography of the *n*-hexane fraction, where a mixture of chloroform and *n*-hexane was used as eluent. Subfraction F6 was obtained from column chromatography of combined ethyl acetate and chloroform fractions. The F1 subfraction was of yellow color, while F2–F5 were green, and F6 was red. Antimicrobial activity of these subfractions was checked against variety of bacterial strains (*Shigella flexeneri*, *Escherchia coli*, *Bacillus subtilis*, *Pseudomonas aeruginosa*, *Salmonella typhi*, *and Staphylococcus aureus*) and different fungal strains: *Trichophyton longifusus*, *Candida albicans*, *Aspergillus flavus*, *Microsporum canis*, *Fusarium solani*, and *Candida glabrata*. Imipenem was used as the standard antibacterial drug. All subfractions were found inactive against all bacterial strains. All subfractions were found active against *C. albicans*, *F. solani*, *M. canis* except F5. Subfractions F1, F2, F3, F4, and F6 showed antifungal effects against *M. canis* with percent inhibitions of 20, 10, 20, 30, and 40 respectively, while subfractions F2, F3, and F4 presented a frail fungicidal propensity against *F. solani* through the exhibition of percent inhibitions of 5, 7, and 10%, respectively. The percent antifungal propensity of F6 was 30% against *C. albicans* and 40% against *M. canis.* Miconazole was used as standard drug in antifungal propensity. *C. albicans* and *M. canis* are both human pathogens and are responsible for human mycosis. *Candida albicans* causes candidiasis, which is a severe, subacute, or prolonged infection across different parts of the human body that are vulnerable to this infection [41,42], whereas *Microsporum canis* is considered to be liable for the simple ringworm in pets as well as tinea capitis in humans [43]. Different scientists reported the antimicrobial effect of *V. odorata* against different fungal and bacterial strains with potential growth inhibition [44,45].

### 5.2. Antioxidant Activity

Oxidants/free radicals are species that are produced due to various reactions (metabolic and catabolic) taking place in human body. These species cause many degenerative disorders in the human body as cancer, atherosclerosis, etc. Antioxidants are species that trap the free radicals and neutralize their effect. The flavonoid contents were quantified in various solvents, i.e., ethyl acetate (65.36 mg/g), methanol (39.0 mg/g), chloroform (63.89 mg/g), butanol (28.0 mg/g), and aqueous (6.86 mg/g) fractions, while the potential quantity of phenolic contents were also found in chloroform (62.0 mg/g), methanol (34.0 mg/g), ethyl acetate (64.13 mg/g), butanol (28.32 mg/g), and aqueous (6.46 mg/g) fractions of *V. betonicifolia* whole-plant. The chloroform and ethyl acetate fractions have been approved as a potential fount of these phytochemicals. The crude methanolic extract, chloroform, and ethyl acetate fractions of whole-plant *V. betonicifolia* have been reported as potent sources of antioxidants because of the presence of phenolic compounds [33]. 

All samples were evaluated for their antioxidant propensity in terms of the percentage of DPPH free radical scavenging (IC_50_) at different concentrations levels (20, 40, 60, 80, 250, and 500 ppm), of which the results demonstrated concentration-dependent DPPH scavenging activity. The descending pattern of various fractions for the DPPH radical scavenging propensities: chloroform > ethyl acetate > methanol > *n*-butanol > aqueous > *n*-hexane fractions. The demonstration of experimental scavenging potential against the DPPH free radical by these extracts/fractions might be because of the existence of natural compounds that have the ability and potential to display effective and imperative antioxidant propensity. The IC_50_ values of DPPH radicals scavenging for the chloroform, ethyl acetate, and methanol were 80, 82, and 110 μg/m, respectively, while the *n*-hexane fraction showed IC_50_ = 500 μg/mL. All the extracts were found to reveal less free radical DPPH scavenging activity in comparison to the employed standard, BHT. While, in the reducing power assay, because of the occurrence of reductant molecules, the reduction of Fe^+3^ in ferric cyanide takes place via the acceptance of one electron that converts it into the Fe^+2^ ferrous form. A dose-dependent antioxidant effect was observed in all fractions.

Chloroform fraction showed the highest antioxidant effect followed by ethyl acetate, methanol, butanol, *n*-hexane, and aqueous fractions. The chloroform fractions exhibited the maximum activity followed by ethyl acetate and methanolic extracts. The chloroform and ethyl acetate fractions also proved to be effective antioxidants in the reducing power assay. Overall the plant showed significant antioxidant activity [33].

### 5.3. Nematicidal and Larvicidal Activity 

Like human beings plants are also affected through plant pathogenic nematodes. These pathogenic microorganisms destroy fiber crops, horticultural, as well as food crops, declining the yield of crops [46]. A dose- and time-dependent nematicidal effect of *V. betonicifolia* was observed. The crude methanolic extract and its different fractions—chloroform, *n*-hexane, ethyl acetate, butanol, and aqueous—were tested at different concentrations (0.5, 1, and 2%) against *Meloidogyne incognita*, *Meloidogyne javanica*, *Cephalobus littoralis*, *and Helicotylenchus indicus.* The ethyl acetate fraction was found to be extremely effective against *M. incognita*. At 2% dosage, 40% mortality rate was exhibited by the fraction of ethyl acetate followed by chloroform and methanolic extracts, which were displayed 38% and 37% mortality after 24 h, respectively. Whereas, after 48-h, ethyl acetate, chloroform, and methanol extracts displayed percentage mortalities of 77%, 71%, and 60%, respectively, at 2% concentration. The other solvents fractions also presented nematicidal activity at all their dosage levels in contrast to the control treatment. The maximum mortality of *M. javanica* was occurred the chloroform extract, followed by the ethyl acetate and methanolic extracts with percentage mortality of 68%, 65%, and 58%, respectively, after 48 h at 2% concentration. The percentage of mortality of *C. littoralis* with the chloroform and ethyl acetate fractions was 66% and 62%, respectively, while chloroform and ethyl acetate fractions displayed 49% and 57% mortality of *H. indicus*, respectively, after 48 h at the 2% concentration level [33,47].

Dengue fever is considered a major health issue worldwide, especially in tropical countries where promising ecological circumstances are liable for the proliferation of the vector *Aedes aegypti* [48], and it can be hegemonized through slowing the development of *Aedes aegypti*. The larvicidal effect of the *V. betonicifolia* whole-plant methanolic extract and its different fractions (*n*-hexane, chloroform, ethyl acetate, *n*-butanol, and aqueous) was tested against *A. aegypti* at different concentrations (10, 50, 100, 200, and 500 ppm). The chloroform fraction presented excellent mortality against this vector with LC_50_ 13.03 μg/mL, followed by ethyl acetate and methanolic extracts having LC_50_ of 16.00 μg/mL and 61.30 μg/mL [33].

### 5.4. Anthelmintic and Leishmanicidal Activity

Anthelminthic activity of the methanolic extract and different fractions (*n*-hexane, chloroform, ethyl acetate, *n*-butanol, and aqueous) of *V. betonicifolia* whole-plant at concentrations ranging from 25 to 100 mg/mL against *Pheretima posthuma* (an adult earthworm) was checked. The anthelmintic activity of ethyl acetate and chloroform fractions was found significant against *P. posthuma*, in a concentration-dependent manner (100 mg/mL), mortality times were 42 and 58 min, respectively [47]. Piperazine citrate was used as the standard drug and distilled water was used as the control. The larvae of these pathogens infect plants by forming a gall at the roots, affecting the quality and yield of the plants. The ethyl acetate, chloroform, and methanolic extracts of *V. betonicifolia* whole-plant proved effective in controlling the pathogenicity of these plant parasites.

The leishmanicidal activity of the crude methanol extract and different fractions (*n*-hexane, chloroform, ethylacetate, *n*-butanol, and aqueous) of *V. betonicifolia* was checked against *Leishmania major*. None of the tested plant samples showed leishmanicidal activity with IC_50_ more than 100. No leishmanicidal effect was observed against any of the tested samples [49]. 

The leishmanicidal activity of of various subfractions (F1–F6) of whole *V. betonicifolia* plant was checked against *Leishmania major.* None of the subfractions showed any leishmanicidal effect [40]. Amphotericin B and pantamidine were used as standard drugs.

### 5.5. Toxicological Studies

The acute toxicity of *V. betonicifolia* crude methanolic extract and *n*-hexane fraction was carried out to evaluate any possible toxicity. BALB/c mice were treated with different doses (500, 1000, and 2000 mg/kg), while the control group received saline (10 mL/kg). The *V. betonicifolia* methanolic extract and *n*-hexane fraction were found safe at all doses (500, 1000, and 2000 mg/kg i.p). During the 24-h assessment time, test animals were found to be normal [34,50]. Therefore, both of the tested plant extracts were considered safe up to the dose level of 2000 mg/kg.

Muhammad and Saeed [33] reported the phytotoxic and cytotoxic activity of *V. betonicifolia* methanolic extract and its fractions (chloroform, *n*-hexane, ethyl acetate, *n*-butanol, and aqueous fractions) at different concentrations (10, 100, and 1000 ppm). *V. betonicifolia* whole-plant showed significant phytotoxicity against plant *Lemna minor*. The outstanding phytotoxic effects were observed against *n*-butanol fraction with 83% inhibition while the ethyl acetate fraction showed 73% growth inhibition. All fractions were weak phytotoxic beside the aforementioned fraction which have the phytotoxic potential. 

The cytotoxic effect of *V. betonicifolia* against brine shrimps was carried out and percentage mortality of methanol extract and its different fractions was determined. Aqueous (LC_50_ = 46 μg/mL) and chloroform (LC_50_ = 56 μg/mL) fractions showed significant effects. Etoposide was used as standard cytotoxic drug. It is interesting to note that except *n*-hexane (LC_50_ = 60.08 μg/mL), and aqueous fractions, all samples were not effective cytotoxic at concentration of 10 μg/mL. While at 1000 μg/mL concentration, 100% cytotoxicity was shown by methanolic extract and chloroform fraction. 

Phytotoxicity of various subfractions (F1–F6) of *V. betonicifolia* whole-plant against *Lemna minor* [40] was determined. The F6 subfraction showed excellent phytotoxic activity with % growth inhibition 85, 60, and 25 at 1000, 100, and 10 ppm concentrations, respectively. F1 and F3 also showed good phytotoxicity in a concentration-dependent manner. Other subfractions showed a weak phytotoxic effect. Paraquat (3.142 μg/mL) was used as the standard drug. The phytotoxic potential of a plant or compound is very beneficial from an agriculture point of view, as this assay is helpful in screening of herbicidal compounds. By controlling the weeds in crops, the production, quality, and quantity of crops can be increased. Therefore our tested samples F6, F3, and F1 can be used as natural weeds controlling agrophytochemicals [40].

The cytotoxic potential of various subfractions (F1–F6) were measured against *Artemia salina* brine shrimp. Etoposide was used as the standard drug. Subfractions F5 and F6 (LD_50_ = 175.4 and 160.7 μL/mL) showed significant cytotoxic effects. F4 did not show any cytotoxic activity, while F1, F2, and F3 showed a weak cytotoxic effect. As the concentration of the tested subfraction was increased, the death rate of shrimp also increased. [40].

### 5.6. Neuropharmacological Activities

*Viola betonicifolia* (whole-plant) has been used as a sedative in treatment of various nervous disorders in Pakistani traditional medicines. The crude methanolic extract and *n*-hexane fraction of the whole-plant of *V. betonicifolia* was investigated for neuropharmacological properties, such as anxiolytic, muscle relaxant, sleep induction, and sedative, to ascertain its traditional use. Anxiolytic activity was tested using the staircase test, while the muscle relaxing property of the extract was tested in various muscle relaxant paradigms, i.e., chimney test, traction test, rota rod, and inclined plane. In anxiolytic and muscle relaxant tests, *n*-hexane fraction, methanolic extract (0.3, 0.4, and 0.5 g/kg, i.p.), diazepam (1 mg/kg, i.p.), and distilled water (10 mL/kg i.p.) were administered at 30, 60, and 90 min before performing the tests in BALB/c mice. The *n*-hexane fraction and crude methanolic extract showed significant (*p* < 0.05) dose-dependent anxiolytic and muscle relaxant activities. The crude methanolic extract and *n*-hexane fraction was also screened for a sleep-inducing effect in BALB/c mice. For the phenobarbitone sleep induction test, the control animal group was treated with distilled water (10 mg/kg) and the standard animal group was treated with diazepam (4 mg/kg). The methanolic extract and *n*-hexane fraction were employed at 0.3, 0.4, and 0.5 g/kg. After 30 min of treatment, all animals were injected with phenobarbitone sodium 35 mg/kg (i.p.). Each animal was observed for the onset and duration of sleep. The duration of sleep or hypnosis was considered as the loss of postural reflexes. The *n*-hexane fraction and methanolic extract notably (*p* < 0.05) reduced the latency time and increased the total sleeping duration in dose-dependent manner. The duration of sleep in diazepam and distilled water treated groups was 56.45 min and 7.34 min, respectively, while the duration of sleep in the methanolic extract and *n*-hexane fraction-treated groups were 8.13, 13.98, and 25.23 min and 5.13, 18.08, and 30.03 min at doses of 0.3, 0.4, and 0.5 g/kg, respectively (Table 4). The results suggest that methanolic extract and *n*-hexane fraction possessed anxiolytic, muscle relaxant, sleep inducing (sedative), and hypnotic activity and, thus, provided pharmacological justification for the use of this plant as a sedative and for the relief of various nervous disorders [38,50]. Monadi and Rezaie [53] reported the sedation and preanesthetic effects of the leaf extract of *V. odorata* at doses ranging from 100 to 400 mg/kg in rats.

### 5.7. Antipyretic Activity

Since ancient times medicinal plants have played a significant role in curing diseases and relieving physical suffering. Pyrexia, or fever, is the increase in body temperature due to several reasons. Antipyretics are drugs which can reduce elevated body temperature. They inhibit COX-2 expression thereby inhibiting prostaglandin synthesis. Medicinal plants are a good source of antipyretic agents since ancient times because of lower toxicity and no side effects, thus represent an excellent alternative to synthetic drugs [54]. The antipyretic effect of *V. betonicifolia* methanolic extract was checked in BALB/c mice at different concentrations: 100, 200, and 300 mg/kg. Saline at 10 mL/kg was used as the negative control, while paracetamol (150 mg/kg) was used as the standard drug. 

The methaolic extract potentially decreased hyperthermia induced by the yeast. Pyrexia induced by injection of yeast is a useful test for determining antipyretic effects of natural and synthetic drugs. Yeast induces pyrexia by increasing the synthesis of prostaglandins. The inhibition was found to be dose-dependent and remained significant up to 3 h after dose administration. The maximum antipyretic effect (78.23%) was observed at 300 mg/kg concentration, while the antipyretic effect of paracetamol was 90% at 150 mg/kg [34]. 

Muhammad et al., [51] reported the antipyretic effect of the *n*-hexane fraction of *V. betonicifolia* whole-plant induced by yeast in mice. The antipyretic effect of *n*-hexane fraction of *V. betonicifolia* (300 mg/kg) started from the 1st h and remained significant up to the 5th h post-treatment, while at a dose of 200 mg/kg, the antipyretic effect started after the 2nd h of treatment and remained significant up to the 5th h. A weaker antipyretic effect at the dose of 100 mg/kg was also observed. The *n*-hexane fraction at concentration of 300 mg/kg showed potent antipyretic activity—82.50%—in comparison with standard drug paracetamol (150 mg/kg) which showed 85% antipyretic activity. Khattak et al. [55] reported the antipyretic potential of *V. odorata* in rabbits using *n*-hexane, chloroform, and water-soluble extracts. While the *n*-hexane-soluble fraction showed potent results.

### 5.8. Antidepressant Activity

The *n*-hexane fraction of *V. betonicifolia* whole-plant was checked for its antidepressant activity with the help of the forced swimming test (FST), while line crossing in a special box was used for locomotor activity. The FST is commonly used for the determining the antidepressant activity in animal models. However, *n*-hexane extract did not show any antidepressant activity, and the movements of mice were reduced significantly (*p* < 0.05) in locomotor activity (Table 5). The mice were immobile and inactive at a dose level of 0.5 g/kg. Central nervous system (CNS) depressant activity was enhanced in a dose-dependent manner. In animal models, the prolongation in mobility period shows antidepressant activity, while shortening in mobility time shows the central nervous system depression-like state [56]. No antidepressant effect was observed in comparison with standard fluoxetine because all tested doses of the hexane fraction failed to shorten the duration of immobility. The antidepressant effect was similar to the negative control, which showed that the plant extract cannot stimulate the CNS [50].

### 5.9. Anticonvulsant Activity

Epilepsy is third-most common neurological disorder and is characterized by chronic seizures and disturbances in brain functions. Available anticonvulsant drugs control the epileptic seizures efficiently in approximately 50% of patients. Furthermore, the available drugs have several severe side effects that make treatment difficult, so the discovery of a new natural source of anticonvulsant drugs is imperative [57]. The *n*-hexane fraction of *V. betonicifolia* whole-plant was evaluated for its anticonvulsant potential in pentylenetetrazol- (PTZ) and strychnine-induced convulsions in BALB/c mice. The *n*-hexane fraction, at all stages, reduced pentylenetetrazol-induced convulsions more than the control (10 mg/kg) group. Diazepam was used as the standard drug. The *n*-hexane fraction of *V. betonicifolia* were demonstrated the concertation dependent (300, 400, and 500 mg/kg) anticonvulsant effect. The fits phase, involving ear and facial twitching, and the second phase, comprising a wave of convulsions through the body, were protected 100% at all the tested dosages, but the latency time of remaining phases was enlarged. The *n*-hexane fraction of *V. betonicifolia* were exhibited the supreme effect which was (400 and 500 mg/kg), as the latency time for the general clonic-tonic seizure (fifth phase) was augmented up to 25.3 min. After 24 h, the *n*-hexane fraction of *V. betonicifolia* did not cause mortality at any tested dosage level, but it did not reveal protection in the strychnine-induced anticonvulsant test at different doses of 300, 400, and 500 mg/kg i.p., and the mice ultimately died [51].

### 5.10. Analgesic Activity

Pain is a revolting sensation as well as an emotional experience that is essentially linked with genuine or impending tissue impairment and is always a cautionary indication and chiefly defensive in nature, however it frequently produces a number of discomposure that lead to numerous adverse effects. Analgesics are pain relieving agents that act on the central nervous system and peripheral pain mediators to reduce pain without changing consciousness [58,59]. In previous reports, the acetic acid-induced writhing-test, tail immersion test, and hot plate approaches were exploited for the evaluation of analgesic propensity of *V. betonicifolia* whole-plant in BALB/c mice [34]. The methanolic extract and *n*-hexane fraction of *V. betonicifolia* presented a dose-dependent analgesic effect at various doses (100, 200, and 300 mg/kg i.p.). In acetic acid-induced analgesia, both the methanolic extract [34] and *n*-hexane fraction [52] showed maximum inhibition (78.9 and 85.2%, respectively) at 300 mg/kg dose. The inhibition potential of standard drugs diclofenec (96.2%) was superior in comparison to the maximum dosage of methanolic extract as well as *n*-hexane fraction. The methanolic extract and *n*-hexane fraction demonstrated concentration-dependent analgesia in several pain models, i.e., hot plate and tail immersion tests. The hot plate test for the methanolic extract displayed that latency time was meaningfully (*p* < 0.05) augmented from 17.22 to 69.96% at the dosage level of 100 to 300 mg/kg while in the existence of naloxone, the analgesic effect of TramadolR (30 mg/kg) and methanolic extract (200 and 300 mg/kg) was inverted extremely. The hot plate test of *n*-hexane fraction showed that latency time for the mice was augmented at the dosage level of 300 mg/kg. Overall, analgesic activity was low and was not antagonised by naloxone. 

The analgesic effect of the methanolic extract of *V. betonicifolia* was also significant in the tail immersion test. The % inhibition of pain was 17.22, 22.29, and 68.58 at 100, 200, and 300 mg/kg of methanolic extract, respectively. TramadolR, a standard analgesic, displayed noticeable activity (76.73%). However, the *n*-hexane fraction was a weak analgesic at the dose of 300 mg/kg in the hot plate and tail immersion tests.

### 5.11. Anti-Inflammatory Activity

Inflammation is a type of biological response of vascular tissues to harmful stimuli such as pathogens, damaged cells, or irritants. These days many synthetic anti-inflammatory drugs are present in market, but their prolonged use may cause adverse effects on health of human beings due to toxicity. Currently scientists are paying great attention to discover medicinal plants with anti-inflammatory potential, this will lead to the discovery of novel therapeutic agents, and these agents may inhibit inflammation and also be used in disease conditions where the inflammation response enhances the disease process [60]. The anti-inflammatory activity of several plant extracts and isolated compounds has already been reported [61]. The anti-inflammatory effect of whole-plant *V. betonicifolia* methanolic extract and *n*-hexane fraction in BALB/c mice at different doses (100, 200, and 300 mg/kg i.p) has been reported. This activity was also found to be dose-dependent. The injection of the carrageenan in paw created an inflammatory edema which increased gradually. Diclofenac sodium at a concentration of 10 mg/kg was used as the standard drug. The methanolic extract [34] and hexane fraction [52] exhibited anti-inflammatory activity at the dose of 300 mg/kg that became significant after 2 h carrageenan injection and was maintained throughout the experiment with maximum effects of 60.9% and 60.8%, respectively. The extracts (200 and 300 mg/kg) induced significant (*p* < 0.01) effects and the anti-inflammatory effect of diclofenac sodium (10 mg/kg) was greater than that of the both extracts. 

The histamine induced inflammatory edema was significantly inhibited by methanolic extract at 200 and 300 mg/kg doses. The methanolic extract showed a reasonable anti-inflammatory effect in a dose-dependent manner and remained significant up to 5th h of administration [34]. While the histamine induced inflammation was not effected by *n*-hexane fraction [52].

Koocheck et al. [62] reported the anti-inflammatory effect of aqueous extract of *V. odorata*. The plant was partially effective in preventing formalin-induced lung damage.

### 5.12. Prokinetic and Laxative Effects

Prokinetics are important drugs which increase the intestinal motility by enhancing the frequency of contractions in the small intestine but not disrupting their rhythm. They relieve gastrointestinal problems such as abdominal discomfort, nausea, vomiting, bloating, constipation and heartburn, gastritis, and gastroparesis. Laxative substances are used to soften the stool and increase bowel movement. Both laxatives and prokinetics are used to prevent constipation. Laxatives may be administered orally or rectally [63,64].

The laxative and prokinetic effects of *V. betonicifolia* whole-plant have been reported. In vivo studies were carried on BALB/c mice, while for in vitro studies experiments were conducted on gut preparations from guinea pigs and locally bred rabbits. The crude methanolic extract of *V. betonicifolia* whole-plant showed partially atropine-sensitive prokinetic (50 and 100 mg/kg) and laxative (30 and 100 mg/kg) effects in mice models. During in vitro tests in isolated rabbit jejunum and guinea pig ileum, the methanolic extract showed dose-dependent contractions (0.01–0.3 mg/mL and 0.03–5 mg/mL, respectively). The spasmogenic effect was partially sensitive to atropine, while the presence of pyrilamine, SB203186, or hexamethonium had no effect in either gut preparation. Methanolic extract partially inhibited acetylcholinesterase enzyme 19% in the in vitro studies. The spasmodic effect of methanolic extract was more efficacious in guinea pig ileum than rabbit jejunum preparation. This study showed the prokinetic and laxative effects of *V. betonicifolia* in mice, partially mediated through cholinergic action. The in vitro spasmodic effect of the plant extract was also partially sensitive to atropine indicating more than one mechanism in the gut stimulant effect. This study provides evidences for the medicinal use of *V. betonicifolia* in indigestion and constipation [35]. Alcoholic extract of *V. odorata* at 200 mg/kg and aqueous extract at 400 mg/kg showed significant laxative effects [65].

### 5.13. Insecticidal Activity 

Insects cause serious damage to agricultural crops and plants. Since the development of agriculture protection of seeds and stored grains has always been a major problem, natural plant products, especially essential oils, have been reported as potential insecticides, and these natural products can be used to develop environment friendly methods for control of insects [66,67]. To eliminate the toxic effects and pollution of synthetic pesticides, the search for natural insect repellent sources continues [68].

The crude methanolic extract and its different fractions (*n*-hexane, chloroform, ethyl acetate, *n*-butanol, and aqueous) obtained from *V. betonicifolia* whole-plant were screened for insecticidal effect against three different insects: *Tribolium castaneum, Rhyzopertha dominica*, and *Callosobruchus analis.* Permethrin was used as standard insecticidal drug. Samples demonstrated low-to-moderate insecticidal activity. The crude methanolic extract showed 20 and 40% activity against *T. castaneum* and *C. analis*, respectively, while chloroform exhibited 40% mortality against *T. castaneum*. The *n*-hexane fraction showed 40 and 20% mortality against *R. dominica* and *C. analis*, respectively. The ethyl acetate fraction was found inactive against the tested insects. The *n*-butanol fraction showed low mortality (10%) against *T. castaneum*, while moderate mortality (40%) was shown by aqueous against *C. analis* [49]. 

Muhammad et al. [40] reported the insecticidal activity of different subfractions (F1–F6) of *V. betonicifolia* whole-plant against three different insects: *Tribolium castaneum*, *Rhyzopertha dominica*, and *Callosobruchus analis*. Permethrin was used as the standard insecticidal drug. Subfrations F1 and F5 showed 20% mortality, and F2 showed 40% mortality against *T. castaneum* [40], whereas F3, F4, and F6 were found inactive against this insect species. Subfractions F2, F3, F4, F5, and F6 showed potential mortality (40, 20, 40, 20, and 20%) against *R. dominica* respectively, while F1 was found inactive against this insect specie. F1, F3, and F6 showed mild mortality, 40, 20, and 20%, respectively, against *C. analis*, while F2, F3, and F4 were inactive against *C. analis.* In comparison to standard drug, an insecticidal effect with 40% mortality may be considered significant. A moderate insecticidal effect against *T. castaneum* and *R. dominica* was observed. Insects species similar to *Tribolium castaneum*, also known as red beetles, attack stored grain products such as flour, cereals, beans, spices, bakery products, dried pet food, dried flowers, chocolate, nuts, seeds, and even dried museum specimens [69]. *R. dominica* is an internationally known beetle and is considered a primary pest for the destruction of stored grains [70]. 

### 5.14. Diuretic Effect

Medicinal plants protect the mankind from several diseases. Diuretics are tremendously important for the treatment of hypertension and also play an important role in enhancing the effect of antihypertensive drugs. Diuretics give relief from pulmonary congestion and peripheral edema; they decrease plasma volume and this decreases the workload of the heart, oxygen demand, and plasma volume and they also help in decreasing blood pressure [71,72].

The diuretic effect of the methanol extract and *n*-hexane fraction obtained from *V. betonicifolia* whole-plant was screened on BALB/c mice at different dose levels (200, 300, and 400 mg/kg intraperitoneally). Frusemide (10 mg/kg) was used as the standard drug. Methanolic extract of *V. betonicifolia* showed a mild diuretic effect but the results were statistically nonsignificant compared to the standard. The *n*-hexane fraction was devoid of any diuretic effects. In ethnomedicine, *V. betonicifolia* has been reported for its diuretic effect [29]. The mild diuretic effect of *V. betonicifolia* may be due to its use as polypharmacy in previous times [49].

## 6. *V. betonicifolia* in the Cosmetics Industry

Melamine performs a dynamic part in providing fortification to the skin from the ultraviolet (UV) ray-induced damage, and abnormality in melamine production leads to UV-induced skin hyperpigmentation [73], such as the abnormal accumulation or overproduction of melanin that causes different kinds of skin hyperpigmentation diseases including age spots, melisma, and freckles. Therefore, numerous skin depigmenting agents, including kojic acid and arbutin, function as tyrosinase quenchers and are applied as skin whitening agents for the treatment of pigmented skin. However, their uses cause serious kinds of adverse effects, e.g., arbutin produces a probable genotoxic effect [74], while kojic acid is liable to cause pigmented contact dermatitis. Therefore, this is why the thirst to investigate nontoxic and operative skin depigmenting chemical entities based on natural compounds is a chief goal. It has been observed that skin moisturizers are involved in slowing down the skin aging process, relieving skin roughening, and providing moisture to skin to maintain its smoothness [75]. These skin toning effects are due to the presence of natural moisturizing factor (NMF) ingredient production accelerator, filaggrin hydrolysis accelerator, and activators responsible for peptidylarginine deiminase activity. Numerous reports indicate that the *Viola betonicifolia* is also present in the formulations of commercially existing skin care products and in skin whitening cosmetic products, plant extracts of *Viola betonicifolia* provided melanogenesis quenchers agents that inhibit the mechanism of melanogenesis, which is responsible for skin darkness. The cosmetic based on the extract of this plant possess such natural compounds which have ability to inhibit the melamine production, act as anti-inflammatories and provide a moisturizing sense to the skin. Therefore, it is very vigorous and effective for the preclusion and relieving of skin inflammation and skin darkness that result from sunburn, and skin staining. The cosmetic is very active in whitening the skin, and may preclude skin disorders due to UV-rays and provide protection to the skin from dermal stains as well as from wrinkles.

## 7. Volatile Oils Composition of *V. betonicifolia*

Chemical composition of different subfractions of the *V. betonicifolia* plant were studied by GC and GC–MS analysis. Subfractions (F1–F5) were obtained from column chromatography of the *n*-hexane fraction, where a mixture of chloroform and hexane was used as eluent. Subfraction F6 was obtained from the combined fractions of ethyl acetate and chloroform by column chromatography. The F1 subfraction was of yellow color while F2–F5 were green and F6 was red. Chemical composition of these subfractions is presented in Table 6 and Figure 3. Total 57 components were identified from all subfractions. Eleven components were identified in subfraction F1; the main constituents of F1 were methyl 10-methyldodecanate (76.0%), methyl myristate (34.9%), 9-hexadecanoic acid methyl ester (5.9%), and neophytadiene (5.5%). Thirteen constituents were identified in F2. The major components of F2 were neophytadiene (11.5%), arachic alcohol (10.6%), methyl laurate (10.0%), tetradecanol (6.4%), and eicosane (6.2%). The chemical composition of F3 showed the presence of thirteen compounds: arachic alcohol (10.0%), eicosane (16.74%), methyl palmitate (14.79%), methylhexadec-9-enoate (11.92%), 9,12,15- octadecatrienoic acid methylester (11.37%), and *n*-heneicocane (9.45%). Twenty-eight chemical constitutes were identified in F4 subfractions. The abundant components were trimethylpentadecane (10.0%), heneicosane (85.87%), t-butyl-7-methyl-3,5-dioxo-6-octenoate (14.89%), tetramethylhexadecane (9.8%), tritriacontane (8.9%), eicosane (6.32%), and dimethylnonane (6.11%). Fourteen constitutes were identified in F5, consisting of 2-pentadecanone trimethyl (10%), hexadecane (21.74%), di-tertbutylphenol (10.6%), and methyl palmitate (5.17%) as major constitutes. F6 subfractions showed the presence of 1,2-benzenedicarboxylic acid, bis(2methylpropyl) ester (3.77%) and the major component was (1S*,2R*,5R*,7S*)-,2,-Dimethyl-7- ethyl-6,8-dioxabicyclo [3.2.1]-oct-3-ene (10%) [40].

## 8. Conclusions

Medicinal treatments through the exploitation of plant-based natural compounds are widely investigated and numerous plants as well as herbs have been proclaimed already for their employments as analeptic. The higher nutritive value and functional characteristics, e.g., antioxidant, analgesic, antidepressant, antipyretic, anticonvulsant, and anti-inflammatory, linked to this plant recommend its exploitation in nutraceutics, medicines, and pharmaceutics. Henceforth, we corroborated that *V. betonicifolia* could be investigated as a potential source of higher added value compounds for the nutraceutical industry as well as the food industry. However, further studies needed to explore the chemical constituents and their pharmaceutical potential. Further experimentation required to explore uses of this multipurpose plant in cosmetic industry as well.

## Figures and Tables

**Table 1:** Quantitative leaf microscopy.

Morphological Features	Range	Means
Palisade-ratio	9–8.25	8.62 ± 0.23
Stomata number (upper epidermis)	12–19	16.70 ± 0.31
Epidermal cells (upper epidermis)	52–73	60.70 ± 0. 39
Stomata number (lower epidermis)	54–62	55.23 ± 0.28
Epidermal cells (lower epidermis)	142–154	140.12 ± 0.43
Vein islet number	7.5–10	8.40 ± 0.25
Veinlet termination number	5–6	5.50 ± 0.11

Data Source: Muhammad et al., [23].

**Table 2:** Micro- and macronutrients in different parts of *V. betonicifolia.*

**Micronutrients (µg/g)**							
**Plant Parts**	**Lead (Pb)**	**Copper (Cu)**	**Chromium (Cr)**	**Iron (Fe)**	**Manganese (Mn)**	**Nickel (Ni)**	**Zinc (Zn)**
**Leaves**	6.45 ± 0.21	23.90 ± 0.22	40 ± 0.15	320 ± 0.33	14 ± 0.11	---	10 ± 0.15
**Petioles**	5.63 ± 0.23	15.56 ± 0.11	20 ± 0.17	340 ± 0.20	9 ± 0.22	0.50 ± 0.11	34 ± 0.26
**Root**	---	42.67 ± 0.26	67 ± 0.20	295 ± 0.19	3 ± 0.02	---	28 ± 0.02
**Flower**	1.34 ± 0.26	40.89 ± 0.27	50 ± 0.10	220 ± 0.31	27 ± 0.25	1.00 ± 0.17	36 ± 0.22
**Whole Plant**	7.23 ± 0.32	80.45 ± 0.22	35 ± 0.19	245 ± 0.45	60 ± 0.22	1.20 ± 0.30	50 ± 0.25
**Macronutrients (µg/g)**							
**Plant Parts**		**Sodium (Na)**		**Potassium (K)**		**Calcium (Ca)**	
**Leaves**		156.00 ± 0.12		890.00 ± 0.26		132.00 ± 0.12	
**Petioles**		623.00 ± 0.32		325.00 ± 0.11		256.00 ± 0.24	
**Root**		214.00 ± 0.38		235.00 ± 0.40		134.00 ± 0.11	
**Flower**		124.00 ± 0.44		170.00 ± 0.56		200.00 ± 0.38	
**Whole plant**		723.00 ± 0.51		191.00 ± 0.122		500.00 ± 0.32	

Data source: Muhammad et al., [22].

**Table 3:** Pharmacological activities of whole *V. betonicifolia* Plant.

Sr #	Pharmacological Activity	Model	Assay	Extract/Fraction/Compounds	Extraction Technique Used	Outcome/Response	Reference
1	**Antibacterial**	In vitro *E. coli, B. subtilis, S. flexeneri*, *S. aureus, P. aeruginosa, S. typhi*	agar well diffusion method	Methanolic, *n*-hexane, chloroform, ethyl acetate, *n*-butanol, aqueous	Maceration and fractionation at room temp.	chloroform fraction showed zone of inhibition against *E. coli* = 20 mm *S. typhi* = 17 mm while Ehylacetate fraction showed ZOI against *E. coli* = 10 mm; *S. typhi* = 15 mm. *n*-hexane fraction showed no activity	[39]
		*S. flexeneri, E. coli, B. subtilis, P. aeruginosa, S. typhi, S. aureus*	agar well diffusion method	Subfractions F1-F6	Maceration and fractionation at room temp.	No activity observed	[40]
	**Antifungal**	*T. longifus, C. albicans, F. solani, A. flavus, M. Cani, C. galaberata*	Disc diffusion method	Methanolic, n-hexane, chloroform, ethyl acetate, *n*-butanol, aqueous	Maceration and fractionation at room temp.	% inhibition of methanolic extract against *T. longifus* 30%, *M. Cani*; 20%; *n*-Butanol = nil; Chloroform fraction inhibited *M. cani*; 20%; *F. solani* 10%; Ethyl acetate fraction also inhibited growth of different strains, Aqueous fraction inhibited the growth of *C. albicans* (30%) and *M. cani* (40%).	[39]
		*T. longifus, C. albicans, F. solani, A. flavus, M. Cani, C. galaberata*	Disc diffusion method	Subfractions F1-F6	Maceration at room temp.	Potential inhibition effect against all strains was observed	[40]
2	**Antioxidant**	In vitro	Total phenolic contents	ethylacetate, methanol, chloroform, butanol, Aqueous	Maceration and fractionation at room temp.	chloroform = 62.0 mg/g, methanol = 34.0 mg/g, ethyl acetate = 64.13 mg/g, butanol = 28.32 mg/g, aqueous (6.46 mg/g)	[33]
			DPPH radical scavenges assay	ethylacetate, methanol, chloroform, butanol, Aqueous, *n*-hexane		IC_50_ values of different extracts and fractions, chloroform = 80 μg/m, ethyl acetate = 82 μg/m, methanol = 110 μg/m, *n*-hexane IC_50_ = 500 μg/mL, *n*-butanol = 176 μg/m, aqueous = 496 μg/m	
			Total flavonoid contents	ethylacetate, methanol, chloroform, butanol, Aqueous		Ethyl acetate = 65.36 mg/g, methanol = 39.0 mg/g, chloroform = 63.89 mg/g, *n*-butanol = 28.0 mg/g, aqueous = 6.86 mg/g	
3	**Nematicidal**	*M. incognita, M. javanica, C. littoralis* *H. indicus*		methanolic chloroform, *n*-hexane, ethyl acetate, butanol, *n*-aqueous	Maceration and fractionation at room temp.	Dose and time-dependent mortality effect was observed. After 48 h at 2% conc. *M. incognita;* ethyl acetate = 77%, chloroform = 71%, methanol = 60% *M. javanica;* ethyl acetate = 65%, chloroform = 68%, methanolic = 58% *C. littoralis*; Chloroform = 66%, ethyl acetate = 62 *H. indicus;* chloroform 49%, ethyl acetate fractions = 57% mortality	[33,47]
	**Larvecidal**	*Aedes aegypti*		Methanolic extract *n*-hexane, chloroform, ethyl acetate, *n*-butanol, aqueous	Maceration and fractionation at room temp.	Chloroform fraction showed LC_50_ = 13.03 μg/mL, followed by ethyl acetate and methanolic extract 16.00 and 61.30 μg/mL respectively	[33]
	**Anthelmintic**	*Pheretima posthuma* (an adult earthworm)		Methanolic extract *n*-hexane, chloroform, ethyl acetate, *n*-butanol, aqueous	Maceration and fractionation at room temp.	Time and dose-dependent effect was observed	[47]
	**Leishmanicidal**	*Leishmania major*		Methanolic extract *n*-hexane, chloroform, ethylacetate, *n*-butanol, aqueous F1-F6 subfractions	Maceration and fractionation at room temp.	IC_50_ > 100 μg/mL No activity observed No activity observed	[40,49]
4	**Toxicological study**	Phytotoxic activity	*Lemna minor*	Methanolic extract *n*-hexane, chloroform, ethyl acetate, *n*-butanol, aqueous	Maceration and fractionation at room temp.	The outstanding phytotoxic effects were observed against *n*-butanol fraction with 83% inhibition while the ethyl acetate fraction showed 73% growth inhibition. Weak effect was observed by other fractions.	[33]
				F1-F6 subfractions		F1, F3, and F6 showed concentration-dependent phytotoxic effect. All other subfractions showed weak effect.	[40]
		Cytotoxic activity	Brine shrimp lethality assay	Methanolic extract *n*-hexane, chloroform, ethylacetate, *n*-butanol, aqueous	Maceration and fractionation at room temp.	Concentration-dependent effect was observed. Aqueous = LC_50_ 46 μg/mL and chloroform = LC_50_ 56 μg/mL *n*-hexane = LC_50_, 60.08 μg/mL	[33]
				F1–F6 subfractions		F5 and F6 (LD_50_ = 175.4 and 160.7 μL/mL) showed significant cytotoxic effects. F4 did not showed any cytotoxic activity while F1, F2, and F3 showed weak cytotoxic effect.	[40]
5	**Neuropharmacological**	In vivo BALB/c mice Anxiolytic activity	Staircase test	methanolic extract *n*-hexane fraction	Maceration and fractionation at room temp.	Significant Dose-dependent effect observed	[38,50]
		In vivo BALB/c mice muscle relaxant	Chimney test, Traction test, Rota rod, and Inclined plane			Significant Dose-dependent effect observed	
		In vivo BALB/c mice sleep induction	Hypnotic test and sedative test			*n*-hexane fraction and methanolic extract notably reduced the latency time and increased the total sleeping duration in dose-dependent manner	
6	**Antipyretic**	In vivo BALB/c mice	Brewers-induced pyrexia	Methanol extract *n*-hexane fraction	Maceration and fractionation at room temp.	methaolic extract = 78.23%, *n*-hexane = 82.50% at dose 300 mg/kg	[34,51]
7	**Antidepressant**	In vivo BALB/c mice	Forced swimming test (FST)	*n*-hexane fraction	Maceration and fractionation at room temp.	No antidepressant effect	[50]
			locomotor activity by line crossing test			No antidepressant effect	
8	**Analgesic**	In vivo BALB/c mice	Acetic acid induced writhing test	Methanolic extract *n*-hexane fraction	Maceration and fractionation at room temp.	In acetic acid induced analgesia, methanolic extract and *n*-hexane fraction showed the maximum inhibition 78.9% and 85.2% at 300 mg/kg dose.	[34,52]
			Tail immersion test			Significant analgesic effect observed	
			Hot plate test			Significant analgesic effect was observed	
9	**Anticonvulsant**	In vivo BALB/c mice	PTZ induced seizures	n-hexane fraction	Maceration and fractionation at room temp.	Dose-dependent effect observed. No mortality observed	[51]
			strychnine induced convulsions			No activity, all mice died	
10	**Anti-inflammatory**	In vivo BALB/c mice	carrageen induced edema Histamine induced edema	Methanolic extract *n*-hexane fraction	Maceration and fractionation at room temp.	Dose-dependent effect was observed Methanolic extract = 60.9%; *n*-hexane = 60.8% at 300 mg/kg dose. Methanolic extract showed anti-inflammatory effect in a dose-dependent manner. *n*-hexane fraction showed no anti-inflammatory effect	[34,52]
11	**Diuretic**	In vivo BALB/c mice		Methanolic extract *n*-hexane fraction	Maceration and fractionation at room temp.	Methnolic extract showed mild diuretic effect but statistically nonsignificant *n*-hexane fraction showed no activity	[49]
12	**Prokinetic and laxative effects**	In vivo BALB/c mice	spasmogenic effect	Methanolic extract	Maceration and fractionation at room temp.	The crude methanolic extract showed partially atropine-sensitive prokinetic (50 and 100 mg/kg) and laxative (30 and 100 mg/kg) effects	[35]
		In vitro studies on isolated rabbit jejunum and guinea pig ileum				Methanolic extract showed dose-dependent contractions. The spasmodic effect of methanolic extract was more efficacious in guinea pig ileum than rabbit jejunum preparation.	
13	**Insecticidal**	In vitro *Tribolium castaneum, Rhyzopertha dominica*, and *Callosobruchus analis*		methanolic extract *n*-hexane, chloroform, ethylacetate, *n*-butanol, aqueous	Maceration and fractionation at room temp.	Methanolic extract showed 20 and 40% activity against *T. castaneum* and *C. analis,* chloroform fraction showed 40% mortality against *T. castaneum*. *n*-hexane fraction showed 40 and 20% mortality against *R. dominica* and *C. analis*. Ethyl acetate fraction showed no activity while aqueous fraction showed 40% effects against *C. analis*	[49]
				subfractions (F1–F6)		significant effect observed	[40]

**Table 4:** Effect of methanolic extract and n-hexane fraction on phenobarbitone-induced sleep in mice.

Treatment	Dose	Onset of Sleep (Min)	Duration of Sleeping (Min)
Distilled water	10 mL/kg	25.12 ± 1.25	7.34 ± 2.28
Diazepam	4 mg/kg	5.45 ± 0.08	56.45 ± 0.00
*n*-hexane fraction	0.3 g/kg	30.45 ± 1.97	5.13 ± 0.99
	0.4 g/kg	13.79 ± 1.98	18.08 ± 0.76
	0.5 g/kg	9.08 ± 1.01	30.03 ± 1.98
Methanolic extract	0.3 g/kg	23.45 ± 0.87	8.13 ± 0.97
	0.4 g/kg	15.78 ± 0.78	13.98 ± 1.76
	0.5 g/kg	10.98 ± 0.91	25.23 ± 1.46

All animal groups were treated with phenobarbitone (35 mg/kg). Data presented as mean ± SEM (n = 6).

**Table 5:** Antidepressant activity of *n*-hexane fraction of *V. betonicifolia* whole-plant.

Treatment	Dose	Immobility Time (s)
Distilled water	10 mL/kg	110 ± 0.09
*n*-hexane fraction	0.3 mg	157 ± 1.07
	0.4 mg	206 ± 1.72
	0.5 mg	215 ± 0.93
Fluoxetine	15 mg	30.34 ± 0.00

Values represent the time of immobility (s) in the forced swimming bath for 6 min. Data presented as mean ± SEM (n = 6).

**Table 6:** Chemical composition of volatile oils (subfractions F1–F6) of *V. betonicifolia*.

Sr#	Compound	% Area						
		F1	F2	F3	F4	F5	F6	Retention Time
1	3-Hexanone	-	-	-	1.48	-	-	4.48
2	2-Hexanone	-	-	-	2.01	-	-	4.59
3	Heptane	-	-	-	3.35	-	-	5.38
4	Dimethyl-1-heptene	-	-	-	2.68	-	-	5.9
5	Octane-4-methyl	-	-	-	1.6	-	-	6.5
6	4-Methyldecane	-	-	-	1.4	-	-	10.74
7	Dodecane	-	-	-	1.03	-	-	11.95
8	Dimethylnonane	-	-	-	6.11	-	-	12.1
9	1-Decanol	-	-	-	1.7	-	-	12.5
10	Tricosane	-	-	-	1.03	-	-	13.2
11	3,8-Dimethyl undicane	-	-	-	1.5	-	-	17.11
12	Pentadecane	-	-	-	1.8	2.7	-	17.4
13	4,6-Dimethyldodecane	-	-	-	1.99	-	-	17.9
14	1-Octadecanol	-	-	-	1.46	-	-	18.09
15	Tetradecane	-	-	-	2.2	1.15	-	20.15
16	Tetracosane	-	-	-	1.03	-	-	21.5
17	Docosane	-	-	-	1.7	-	-	22.3
18	Cyclohexadecane	-	-	4.35	3.2	-	-	22.5
19	2,4-Di-tert-butyl phenol	4.13	3.76	-	-	10.6	-	22.51
20	Tritriacontane	-	-	-	8.9	-	-	22.74
21	Octylether	-	-	-	2.6	-	-	22.90
23	Tetramethylhexadecane	-	-	-	9.8	-	-	23.27
24	Tetradecanol	-	6.42	-		4.3	-	24.06
25	Hexadecane	-	3.15	1.89	1.5	21.74	-	24.2
26	Nonadecane	-	-	-	2.78	-	-	24.8
27	Trimethylpentadecane	-	-	-	100	-	-	25.1
28	t-Butyl 7-Methyl-3,5-dioxo-6-octenoate	-	-	-	14.89	-	-	25.27
29	Oxalic acid, 6-ethyloct-3-yl isobutylester	-	-	-	4.61	-	-	27.0
30	Eicosane, 3-phenyl	-	-	-	1.75	-	-	27.32
31	9-Methylene-fluorene	-	-	-	1.17	-	-	27.32
32	*n*-Pentadecanol	1.41	-	-	-	-	-	27.73
33	Octadecane	-	2.5	2.24	-	1.43	-	27.85
34	Methyl laurate	-	10		-		-	28.3
35	Neophytadiene	5.54	11.52	1.11	-	2.39	-	28.53
36	2-Pentadecanone trimethyl	-	-	-	10	-	-	28.46
37	Hexahydrofarnesyl acetone	-	-	1.09	-	-	-	28.64
38	2-Benzenedicarboxylic acid, bis(2methylpropyl) ester (1S*,2R*,5R*,7S*)-,2,-Dimethyl-	-	-	-	-	-	3.77	29.07
39	9-Hexadecanoic acid, methyl ester	5.96	-	-	-	-	-	29.63
40	Methylhexadec-9-enoate	-	-	11.92	-	-	-	29.64
41	Methyl-palmitoleate	-	2.88	-	-	2.5	-	29.92
42	Methyl palmitate	-	-	14.79	-	5.17	-	29.98
43	Methyl 10-methyldodecanate	10	-	-	-	-	-	29.98
44	Methyl tridecanoate	-	1.44	-	-	-	-	30.04
45	1 Eicosanol	1.15	-	-	-	-	-	31.06
46	Chlorphyrifos	-	2.79	-	-	-	-	31.13
47	Eicosane	-	6.17	16.74	6.32	2.24	-	31.16
48	2- Ethyl-1-dodecene	-	-	3.2	-	-	-	31.29
49	Methyl myristate	34.94	3.65	-	-	-	-	31.58
50	Methyl linoleate	4.7	-	-	-	-	-	32.65
51	Methyl oleate	3.62	1.63		-	-	-	32.73
52	9,12,15-Octadecatrienoic acid methylester	-	-	11.37	-	-	-	32.77
53	Methyl pentadecanoate	1.34	-		-	-	-	33.12
54	1-Tridecanol		-	1.1	-	-	-	33.51
55	Arachic alcohol	3.15	10.6	10.0		-	-	34.11
56	n-Heneicocane	-	-	9.45	85.87	-	-	34.18
57	7-ethyl-6,8 dioxabicyclo [3.2.1]-oct-3-ene	-	-	-	-	-	10	38.96

## Introductory Note about Corresponding Author

**Dr. Komal Rizwan** is an Assistant Professor in Department of Chemistry, Government College Women university Faisalabad, Pakistan. She earned her Ph.D. in chemistry from Government college University Faisalabad Pakistan in 2016 and also carried out her research work at University of Pennsylvania, Philadelphia, Pennsylvania United States. She has numerous academic publications in national and international journals of high repute to her credit. Her research interests include Natural product chemistry, Organic Synthesis and Medicinal Chemistry.
